# Posterior tracheal diverticulum: a case report

**DOI:** 10.1186/s13256-024-04851-2

**Published:** 2024-10-31

**Authors:** Afsaneh Safarian, Mehdi Karimi, Niloofar Deravi, Reza Naseri, Khosrow Agin

**Affiliations:** 1 Department of Internal Medicine, Loghman Hakim Hospital, Shahid Beheshti University of Medical Science, Tehran, Iran; 2 Faculty of Medicine, Bogomolets National Medical University, Kyiv, Ukraine; 3 School of Medicine, Shahid Beheshti University of Medical Science, Tehran, Iran

**Keywords:** Diverticulum, Tracheal diverticulum, Tracheal, Case report, Literature review

## Abstract

**Background:**

Tracheal diverticulum is a rare condition often linked to other malformations. This case study highlights the posterior tracheal diverticulum, covering its causes, symptoms, diagnosis, treatment, and prognosis. The report is significant due to the rarity of tracheal diverticulum and potential for misdiagnosis, which can result in complications such as respiratory infections. The case offers novel insights into the presentation and management of tracheal diverticulum, helping to guide future diagnosis and treatment.

**Case presentation:**

A 73-year-old Iranian man with a history of cardiac surgery 15 years ago was admitted to the Loghman Hakim Hospital in Tehran, Iran, for retrosternal chest pain, shoulder radiation, and a persistent cough lasting 4 months. The patient underwent cardiac tests and a lung computed tomography scan, which showed a 16 × 18 mm air-filled outpouching connected to the trachea’s right posterolateral side, leading to a diagnosis of tracheal diverticulum. The patient was treated with bronchodilators and antibiotics.

**Conclusions:**

Tracheal diverticulum is typically asymptomatic but can present with respiratory difficulties, dysphagia, and hoarseness. Diagnosis relies on imaging, and treatment ranges from conservative management to surgical intervention, particularly in symptomatic cases or those with complications. Recognizing tracheal diverticulum in surgical and anesthesia planning is crucial to prevent severe risks such as airway obstruction or trauma. This case report highlights the importance of early detection and personalized management, potentially improving patient outcomes and guiding clinical decision-making in similar cases.

## Introduction

Tracheal diverticulum (TD) is a rare anatomical disorder where an outpouching occurs from the tracheal wall, which may be congenital or acquired. It is typically discovered incidentally and can manifest with varying symptoms or be asymptomatic [1, 2].

This study presents a case of posterior TD, explores the literature on this rare entity, emphasizes its clinical significance, and underscores the importance of understanding, researching, and enhancing patient outcomes for this condition.

## Case report

### Case presentation

A 73-year-old Iranian man was admitted to Loghman Hakim Hospital in Tehran, Iran, with retrosternal chest pain spreading to his shoulders and a productive cough for 6 months. He underwent mitral valve repair and coronary bypass surgery 15 years ago, with no history of smoking or alcohol use. He did not present with fever, hoarseness, difficulty swallowing, or stridor.

### Diagnosis

The patient had typical vital signs and no abnormalities in the neck. Metallic sounds were heard from the mitral valve during cardiac auscultation. The lung auscultation was clear, with no abnormal breath sounds. The rest of the examination was normal.

He underwent cardiac investigations for suspected chest pain and a lung computed tomography (CT) scan for chronic cough. The thoracic CT scan revealed a 16 × 18 mm air-filled outpouching near the tracheal lumen on the right side. Bacteriologic studies were negative for acid-resistant and common bacteria, and spirometry showed no signs of obstructive or restrictive patterns.

**Fig. 1:**
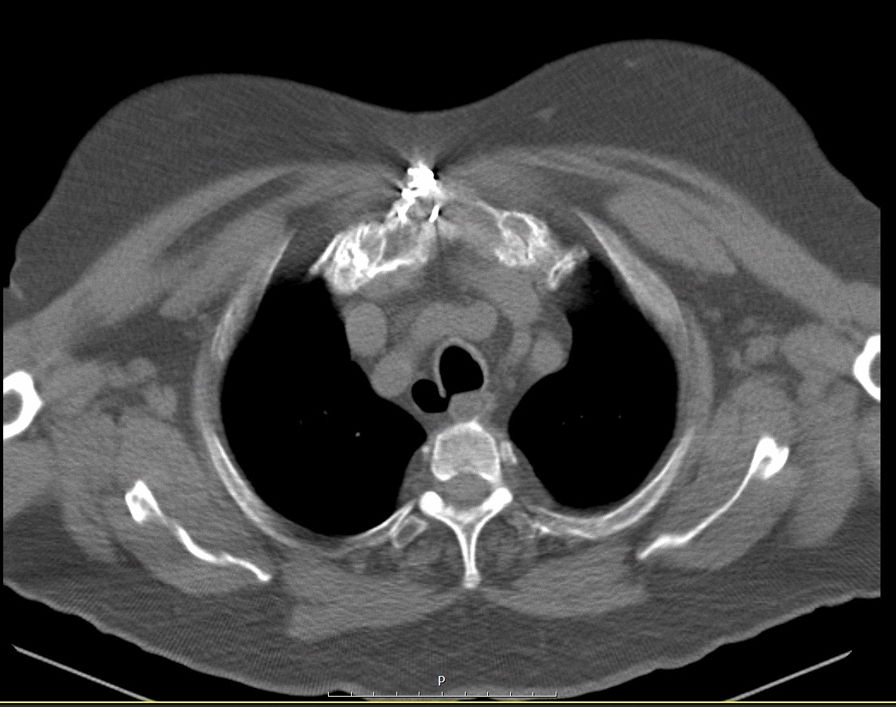
Axial bone window of chest computed tomography (CT) scan

Figure 1 demonstrates the axial bone window of the chest CT scan. There is 16 × 18 mm air-filled outpouching in the proper posterolateral aspect of the trachea at the thoracic inlet, which favors the tracheal diverticula.

### Treatment

The TD was diagnosed after cardiac issues were ruled out. Based on age and medical history, the patient was treated conservatively with bronchodilators, mucolytics, respiratory physiotherapy, and antibiotics, which improved symptoms.

### Patient consent (ethical approval)

The patient provided written informed consent for the publication of this case and any accompanying images. Approval was obtained from the local Institutional Review Board and the Ethics Committee of Loghman Hakim Hospital, Shahid Beheshti University of Medical Sciences (SBUMS), and the study was conducted in accordance with ethical standards.

## Review of tracheal diverticulum

TD is a rare condition in which an outpouching occurs in the tracheal wall, either congenital or acquired. It is usually found incidentally and can cause various symptoms when active [1, 2]. The first recorded case of TD was reported by Rokitansky in 1838 [3].

Most cases of TD present asymptomatically and are often detected incidentally during imaging studies performed for unrelated reasons [4], and may not require treatment if they are not causing any symptoms. While relatively uncommon, this condition can lead to different respiratory and digestive symptoms, making it essential to understand its causes, diagnostic evaluation, and treatment options [1, 5]. They can be found in ~1% of patients in autopsy series. The highest occurrence of TD has been noted among individuals in their fourth decade of life [4].

This article reports a case of posterior TD and explores its causes, risk factors, symptoms, diagnostic methods, treatment options, complications, and long-term prognosis for affected individuals. By delving into these aspects, we aim to provide a comprehensive overview of this condition for medical professionals and individuals seeking information on posterior TD.

### Etiology and risk factors

TD can be congenital or, more commonly, acquired. Congenital TD is caused by faulty tracheal cartilage and consists of respiratory epithelia, smooth muscle, and cartilage (true diverticula). They are smaller, produce more respiratory secretions, and have a narrower mouth. The common site is on the right side, around 4–5 cm below the vocal cords or immediately above the major carina [6]. Acquired TD results from increasing intraluminal pressure, which causes herniation of tracheal sections lacking cartilaginous rings. They consist of ciliated-columnar epithelia that lack smooth muscle and cartilage (pseudo-diverticula). They are more prominent, have a broad mouth, and can start at any level (most commonly on the posterolateral portion of the trachea) [7]. Additionally, they may be caused by cystic distension and thickening of mucous gland ducts [8, 9].

TD risk factors include actions or conditions that may elevate intraluminal pressure within the trachea, weaken its wall, or cause structural abnormalities. Among the risk factors linked to TD development are chronic coughing, chronic obstructive pulmonary disease (COPD), compromised tracheal structures, elevated intraluminal pressure resulting in tracheal wall herniation, cystic distension, thickening of mucous gland ducts, and factors related to previous surgeries [10]. A case report on tracheobronchial diverticulum highlights its potential risk for diving due to the theoretical higher risk of barotrauma during decompression, which can lead to complications such as pneumomediastinum, hemorrhage, or arterial gas embolism [11].

### Clinical presentations

The clinical manifestations of a posterior TD can vary depending on factors such as its size, location, and whether it is congenital or acquired. Most cases of TD present without symptoms and are frequently detected coincidentally during imaging studies conducted for unrelated purposes [4]. The symptoms of TD are primarily respiratory rather than digestive. In symptomatic patients, typical symptoms may include persistent chronic cough, stridor, dyspnea, globus pharyngeus, discomfort, or pain in the neck region. Some cases may involve atypical presentations such as hoarseness, dysphagia or odynophagia, hemoptysis, episodes of hiccups and burping, and choking [5, 9, 12–16].

### Complications and potential risks

Complications are inevitable in individuals with tracheal diverticula, and their severity can vary based on factors such as size, location, and overall health status. In severe and complex cases, these complications can exacerbate the patient’s condition and prognosis [9, 17]. Complications may encompass infection, aspiration, airway obstruction, vascular rupture leading to bleeding, and recurrent respiratory infections. In rare instances, more severe complications such as fistula formation, tracheoesophageal or tracheomediastinal fistulas, or tracheal stenosis may occur [17, 18]. Infected tracheal diverticula can escalate to paratracheal abscess. Additionally, TD may induce dysphonia due to recurrent paralysis from direct compression, potentially complicating tracheal intubation [5, 9]. Recurrent laryngeal nerve paralysis is a possible consequence, manifesting with symptoms such as dysphonia [18, 19].

Table 1 presents a summary of common clinical presentations and complications of patients with TD.

**Table 1:** Common clinical presentations and complications of posterior tracheal diverticulum (PTD)

Clinical presentations	
Typical	Persistent chronic cough, stridor, dyspnea, globus pharyngeus, discomfort or pain in the neck region
Atypical	Hoarseness, dysphagia or odynophagia, hemoptysis, hiccups, burping, choking
Respiratory symptoms	Chronic cough, dyspnea, stridor, recurrent airway infections, hemoptysis, choking
Digestive symptoms	Dysphagia, odynophagia, hiccups, burping
Complications	Infection, aspiration, airway obstruction, rupture of blood vessels and bleeding, recurrent respiratory infections fistula formation, tracheoesophageal or tracheomediastinal fistulas, tracheal stenosis, dysphonia

### Diagnostic evaluation and imaging techniques

Specific signs and symptoms related to this condition need to be assessed during the physical examination of a patient with a TD. In the course of the examination, an individual may observe such signs and symptoms as chronic coughing, episodic respiratory infections, difficulty breathing, and sometimes an evident mass in the area around the neck [9]. In patients with persistent respiratory symptoms after management of gastroesophageal reflux and tracheomalacia, a diagnosis of TD should be considered early [20]. Such imaging techniques as chest radiography, computed tomography (CT) scans, magnetic resonance imaging (MRI), or fiberoptic bronchoscopy are often used in diagnosing TD, especially because they help establish how far the diverticulum is attached to the tracheal lumen [9, 21, 22]. These imaging techniques can demonstrate that there exist air-filled multiple cysts within the tracheal wall, which signifies the presence of a diverticulum [22]. Thin-section multidetector computed tomography (MDCT) is a valuable tool for diagnosing TD [9]. Axial, coronal, and sagittal reformat multiplanar images obtained through MDCT can effectively illustrate the relationship between the diverticulum and the tracheal lumen. While bronchoscopy is another diagnostic option for TD, it may not readily reveal the connection between the diverticulum and the tracheal lumen [9]. MRI is functional in diagnosing TD and monitoring therapeutic efficacy, especially in infected cases [23].

### Treatment options and management strategies

Treatment options vary depending on the size and location of the diverticulum, ranging from observation to surgical intervention. It is essential to individualize the treatment approach based on the patient’s clinical presentation and needs. The choice between conservative and surgical treatment depends on factors such as the patient’s age, physical condition, clinical presentation, the severity of symptoms, and the presence of comorbidities [9, 24]. Conservative approaches are often preferred in treating TD, especially for asymptomatic patients. Conservative management typically involves medical treatment with antibiotics, mucolytic agents, and physiotherapy. This approach aims to prevent infection of the diverticulum and manage symptoms without invasive procedures [9].

Surgical intervention is usually reserved for symptomatic cases where conservative measures have not been effective or when there are specific indications for surgery. Endoscopic cauterization with laser or electrocoagulation can also be considered for symptomatic patients [26].

Surgical treatment of TD involves various approaches depending on the patient’s symptoms and the characteristics of the diverticulum. Symptomatic diverticula can be managed surgically through transcervical resection or endoscopic procedures using laser surgery or electrosurgery. Transcervical repair involves surgical resection of the diverticulum, which is considered a safe and effective treatment option for symptomatic cases [25]. Endoscopic techniques, such as laser surgery or electrocoagulation, can also be utilized for treating TD, especially in cases where conservative measures have not been successful or when there are specific indications for surgery [26].

Bronchoscopic management of TD using argon plasma coagulation (APC) is a minimally invasive approach that effectively treats the condition. APC applies thermal energy to ablate and coagulate the diverticulum tissue, showing promise in cases where conservative measures fail, or surgery is not necessary. Studies confirm APC's efficacy in treating symptomatic cases, establishing it as a safe and efficient treatment option [27, 28].

### Posttreatment care

Ensuring proper posttreatment care for TD is vital for achieving optimal recovery and avoiding complications. Following surgical or bronchoscopic interventions, patients may need personalized care guidance, encompassing monitoring, medication management, dietary adjustments, physical activity recommendations, symptom vigilance, lifestyle modifications, and potential rehabilitation [25, 26].

### Prognosis and long-term outlook

The prognosis and long-term outlook for TD diverticulum are typically favorable, particularly in asymptomatic instances. Symptomatic cases can also fare well with early diagnosis and proper management, leading to favorable outcomes. Timely reporting of new symptoms or complications is essential for prompt intervention. In general, with accurate diagnosis, personalized treatment plans, and adherence to posttreatment care, patients with TD can anticipate a favorable long-term prognosis and quality of life [9, 21].

## Discussion

This case report highlights the importance of recognizing TD, a rare condition that often presents asymptomatically and is typically discovered incidentally during imaging [1, 2]. In this case, the patient’s persistent cough and chest pain led to the identification of a posterior TD through a thoracic CT scan. The conservative management approach, including bronchodilators and antibiotics, was chosen based on the patient’s age, medical history, and relatively mild symptoms. This case underscores the need for heightened awareness of TD, especially in patients with unexplained respiratory symptoms, as early diagnosis and appropriate management can prevent complications such as infection or airway obstruction. Furthermore, it illustrates the significance of considering TD in the context of surgical and anesthesia planning, where unanticipated complications could arise if the condition goes undetected. Overall, while TD is rare, its potential impact on patient health warrants careful consideration in clinical practice, particularly in symptomatic cases.

## Conclusions

TD is an uncommon and rare condition frequently discovered incidentally, as it often presents without symptoms. However, when symptoms do manifest, they can encompass respiratory difficulties, dysphagia, and hoarseness, typically resulting from compression of adjacent structures. Diagnosis predominantly relies on imaging techniques. Management approaches may vary from conservative measures to surgical excision, particularly in symptomatic patients or when complications arise. Considering this condition in surgical and anesthesia planning is crucial to mitigate the risk of severe complications.

## Data Availability

Availability of supporting data.

## Abbreviations

TD: Tracheal diverticulum
PTD: Posterior tracheal diverticulum
CT: Computed tomography
MRI: Magnetic resonance imaging
MDCT: Multidetector computed tomography
